# Larvicidal Evaluation against *Aedes aegypti* and Antioxidant and Cytotoxic Potential of the Essential Oil of *Tridax procumbens* L. Leaves

**DOI:** 10.1155/2021/2172919

**Published:** 2021-01-12

**Authors:** Lethicia B. Brandão, Lizandra L. Santos, Rosany L. Martins, Alex B. L. Rodrigues, Erica de M. Rabelo, Allan K. R. Galardo, Sheylla S. M. da S. de Almeida

**Affiliations:** ^1^Laboratory of Pharmacognosy and Phytochemistry-Federal University of Amapá-10 Highway Jucelino Kubistichek, Km-02. Garden Zero-CEP: 68.902-280, Macapá, AP, Brazil; ^2^Laboratory of Entomology Madical do Institute de Sciense Scientists and Technicians of the State of Amapá (IEPA), Highway Jucelino Kubistichek, Km-10. Fazendinha–CEP: 68.903-419 /68.903-197, Macapá, AP, Brazil

## Abstract

The present study evaluated the antioxidant, cytotoxic, and larvicidal potential of the essential oil of *Tridax procumbens* leaves, as well as identified the compounds present in the essential oil. The antioxidant activity was evaluated by the sequestration method of 2,2-diphenyl-1-picrylhydrazyl radical, the cytotoxic activity was evaluated using *Artemia salina*, the larvicidal bioassay was performed with larvae in the third stage of development of the *Aedes aegypti* mosquito, and the identification of the metabolites was performed by gas chromatography coupled to the mass spectrometer (GC-MS). The phytochemical oil analysis showed the presence of 20 compounds, with thymol and *γ*-terpinene being the main ones. It presented antioxidant activity with an IC_50_ of 194.51 *μ*g mL^−1^, demonstrating antioxidant activity in the highest concentrations tested. It presented low cytotoxic activity against *A. salina*, with an LC_50_ of 1238.67 *μ*g mL^−1^, demonstrating atoxicity in the concentrations tested. The essential oil presented good larvicidal activity when compared to the literature, with an LC_50_ = 79.0 *μ*g mL^−1^ in 24 hours and LC_50_ of 69.15 *μ*g mL^−1^ in 48 hours. In this way, it was possible to identify that the essential oil of the leaves of *T. procumbens* presented potential for the development of a natural larvicide, as well as antioxidant activity satisfactory to the radical DPPH and low toxicity to *A. salina*.

## 1. Introduction

Currently, there are three infectious diseases transmitted by the same vector, the *Aedes aegypti* mosquito, which are circulating at the same time in Brazil, putting public health on alert; they are known as dengue, chikungunya fever, and Zika disease [1]. The control of this vector can be accomplished through the elimination of larvae reproduction sites, and its biological and chemical control can be performed through the use of insecticides [2].

In view of the operational and economic difficulties generated by the increasing resistance of mosquitoes to synthetic insecticides, alternative methods are becoming more prominent and are becoming more efficient and cheaper since they are obtained from renewable resources that are rapidly degradable and have several substances which act simultaneously, making the resistance of insects to these substances occur very slowly [3]. Many studies seek to evaluate the behavior of this vector against the formulation of products with plant origin, in order to identify species that effectively combat *A. aegypti*.

Several plant species, including those belonging to the Asteraceae family, have been studied and used as raw material by the pharmaceutical industry for the creation and development of natural products, with anticancer activities and natural biodegradable insecticidal components. Botanical insecticides are compounds that result from the secondary metabolism of plants [3]; they compose their own chemical defense against herbivorous insects and possess compounds capable of interfering in basic biochemical processes with physiological and behavioral consequences on insects. This type of insecticide has several advantages such as rapid action and degradation, low to moderate toxicity to mammals, increased selectivity, and low phytotoxicity [2].

The *Tridax* genus has been used to treat different diseases; there are several popular reports that point to the usefulness of this plant to circumvent innumerable diseases. In traditional medicine, species of the genus are used as antidiabetic [3], antifungal [4], repellent [2], and immunological [6]. In addition, it has a healing activity and promotes hair growth [6, 7]. Among the species of the genus *Tridax*, *Tridax procumbens* species presents a potential alternative to combat these vectors, through their studies with oil essential. However, its yield for essential oil is low; in this study, the yield of essential oil was 0.02%.

Considering the use of this species by the population, the potential of the Asteraceae family, and the need for studies aimed at identifying the advantages through biological activities, the present study aimed to evaluate the larvicidal, antioxidant, and cytotoxic potential of the essential oil of *Tridax procumbens*, as well as to chemically identify the compounds present in this plant.

## 2. Materials and Methods

### 2.1. Plant Material

Specimens of *T. procumbens* were collected in Fazendinha district (00° 02′23″S and 51° 06′29″ O) in Macapá-Amapá Municipality. Two samples of the species were deposited in the Amapaense Herbarium (HAMAB) of the Institute of Scientific and Technological Research of the State of Amapá (IEPA) under registration number 019178.

### 2.2. The Obtaining Process of the Essential Oil

The process of obtaining the essential oil (EO) was carried out at the Laboratory of Pharmacognosy and Phytochemistry of the Federal University of Amapá (UNIFAP), where the leaves of the plant species were dehydrated in a greenhouse with air circulation at 36°C. The EO was extracted by hydrodistillation in a Clevenger-type apparatus at 100°C for two hours [8]; it was stored in an amber bottle and cooled to −20°C in the dark.

### 2.3. Qualitative Phytochemical Analysis

The phytochemical evaluation of the essential oil was determined by gas chromatography coupled to the mass spectrometer (GC-MS) in equipment of the Shimadzu brand, model GCMS-QP 5050A, in a DB-5HT column of the J&W Scientific brand, with a length of 30 m, a diameter of 0.32 mm, a film thickness of 0.10 *μ*m, and nitrogen as a carrier gas. Thus, the operating conditions of the gas chromatograph were as follows: internal pressure of the column, 56.7 kPa; split ratio, 1 : 20; the gas flow in the column, 1.0 mL.min^−1^ (210°C); the temperature in the injector, 220°C; temperature in the detector or in the interface (GC-MS), 240°C. The initial column temperature was 60°C, followed by an increase of 3°C min^−1^ up to 240°C; it is held constant for 30 min. The mass spectrometer was programmed to perform readings in a range of 29 to 400 Da, at intervals of 0.5 s, with ionization energy of 70 eV.

1 *μ*L of each sample with a concentration of 10,000 ppm dissolved in hexane was injected. The identification of the constituents was based on the comparison of the Kovats index (IK) and mass spectra of each substance with the literature data.

### 2.4. Determination of the Antioxidant Activity

#### 2.4.1. Sequestration of 2,2-Diphenyl-1-picrylhydrazyl (DPPH)

The quantification of the antioxidant activity performed by the DPPH assay was based on the methodology proposed by Sousa et al. [9], Lopes-Lutz et al. [10], and Andrade et al. [11] with some adaptations to laboratory conditions. Initially, a methanolic solution of DPPH (stock solution) was prepared at a concentration of 40 *μ*g mL^−1^, which was kept under the light. After that, six concentrations (7.81, 15.62, 31.25, 62.5, 125, and 250 *μ*g mL^−1^) of EO and of the positive control (ascorbic acid and gallic acid) were prepared. For the evaluation, 2.7 mL of DPPH stock solution was added to a test tube, followed by the addition of 0.3 mL of the EO solution in different concentrations. Then, the white solution was prepared, this being a mixture of 2.7 mL of methanol and 0.3 mL of a methanol solution of each EO concentration evaluated. After 30 minutes, spectrophotometer readings (Biospectro SP-22) were taken at 517 nm wavelength [12]. The assay was performed in triplicate, and the percentage of antioxidant activity (%AA) was calculated with the following equation [9]:

(1) $$\begin{array}{c} \left(\%\text{AA}\right)=100-\left\{\frac{\left[\left({\text{Ab}}_{\text{sample}}-{\text{Abs}}_{\text{white}}\right)\times 100\right]}{{\text{Abs}}_{\text{control}}}\right\}, \end{array}$$

where Abs_sample_ is the sample absorbance, Abs_white_ is the white absorbance, and Abs_control_ is the absorbance control.

The IC_50_ value was also calculated; it is denoted as the concentration of a sample required to decrease the absorbance at 517 nm by 50%. IC_50_ was expressed in *μ*g mL^−1^.

### 2.5. Cytotoxic Activity

The cytotoxicity of the oil was evaluated against larvae of *Artemia salina* Leach, with adaptations [13]. A solution of 250 mL of synthetic sea salt at 35 g.L^−1^ was prepared. In this, 25 mg of *A. salina* eggs were incubated and exposed in artificial lighting within 24 hours for the larvae hatching (nauplii stage). The nauplii were then separated and placed in a dark environment at room temperature for another 24 hours to reach methanuplion stages. The stock solution was prepared to contain 0.06 g of the essential oil and 28.5 mL of the solution of synthetic sea salt, and 1.5 mL of Tween 80 were added to facilitate solubilization. For the negative control, Tween 80 with solution saline (5%) was used, and potassium dichromate (K_2_Cr_2_O_7_) (1%) was used as the positive control. Subsequently, at the end of the dark period, they were selected and divided into 7 groups with 10 methanuplia in each test tube. In each group were added aliquots of the stock solution (100, 75, 50, 25, and 2.5 *μ*L), and the volume was supplemented to 5 mL with a solution of synthetic sea salt, obtaining solutions with final concentrations of 40, 30, 20, 10, and 1 *μ*g mL^−1^, in triplicates. In the end, the number of nonsurvivors for LC_50_ determination was counted using the SPSS® software probit analysis.

### 2.6. Larvicidal Activity

The larvae of *A. aegypti* used in the bioassays came from the colony maintained in the Medical Entomology Laboratory of the Institute of Scientific and Technological Research of Amapá (IEPA), in the 3rd young stage. The biological tests were conducted in a room (3 m × 4 m) with controlled climatic conditions: temperature of 25 ± 2°C, relative humidity of 75 ± 5%, and a photoperiod of 12 hours.

The methodology used followed the standard protocol of the World Health Organization (WHO) [14, 15] with modification in the test vessel. After preliminary tests, the aqueous solutions were selected at the concentrations of 20, 40, 60, 80, and 100 mg.mL^−1^ and they were presolubilized in Tween 80 at 5%.

The EO of *T. procumbens* (0.09 g) was dissolved in 85.5 mL of distilled water and 4.5 mL of Tween 80; Tween 80 with distilled water (1%) was used as the negative control and the larvicidal esbiothrin as the positive control.

For each repetition of treatment, 25 larvae were used, pipetted to a 100 mL beaker containing distilled water. Then, the larvae were removed from the beaker into the test vessel, thus minimizing the time between the preparation of the first and last samples. The safety of the solvent in the employed concentration was verified, being also present in the replicates of the control. During the experiment, the average water temperature was 25°C. After 24 and 48 hours, the dead larvae were counted, being considered as all those that cannot reach the surface.

### 2.7. Statistical Analysis

Data analysis was performed through analysis of variance (ANOVA) and the Tukey test, aiming to identify the significant differences among averages, using the BioEstat program (Ayres). Differences that presented probability levels less than and equal to 5% (*p* ≤ 0.05) were considered statistically significant. The results were expressed as mean ± standard deviation (SD).

## 3. Results and Discussion

### 3.1. Qualitative Phytochemical Analysis

In the GC-MS analysis of the essential oil of *T. procumbens*, it was possible to identify 20 compounds divided between sesquiterpenes and oxygenated terpenes, according to Table 1. Among the major compounds of *T. procumbens* oil, thymol (48.22%), *γ*-terpinene (15.93%), and *o*-cymene (10.27%) are the most important.

There are few EO studies of the *T. procumbens* species. It is a plant native to the African and Asian continents, found easily in countries like India, Malaysia, and China. In the study by Manjamalai et al. [16], it was possible to identify compounds such as Sabinene hydrate, *α*-phellandrene, *β*-caryophyllene, limonene, and *o*-cymene in *T. procumbens* EO.

In the study, the *Eupatorium ballotifolium* Kunth, belonging to the same family as *T. procumbens*, the family Asteraceae, shows similarity in its majority compound [17]. In the two species, thymol and *o*-cymene were identified among the major compounds of the EOs analyzed. These compounds are mentioned in the literature as promising biological agents in different activities, demonstrating the promising potential of the *T. procumbens* species, which presents about 47% thymol in its composition.

### 3.2. Larvicidal Activity against *Aedes aegypti*

The larvicidal activity of *T. procumbens* EO was tested in five different concentrations: 20, 40, 60, 80, and 100 *μ*g mL^−1^ at 24 and 48 hours, as can be seen in Table 2.

According to Table 2, the concentrations of 80 and 100 *μ*g mL^−1^ presented larvicidal activity after 24 h, with a mortality of greater than 50%; the bioassay determined the LC_50_ of 86.163 *μ*g mL^−1^, with *R*^2^ = 0.936. In 48 hours, EO presented activity at concentrations 60, 80, and 100 *μ*g mL^−1^, the LC_50_ of 76.524 *μ*g mL^−1^, and *R*^2^ = 0.923, resulting a high value when compared to the standard larvicidal esbiothrin, with an LC_50_ of 0.0034 *µ*g·mL^−1^.

In the literature, there are not many studies on essential oil against *A. aegypti* of this plant species. However, its insecticidal effect is confirmed in the literature through the extracts of the species, where it is documented the potential larvicidal activity against *A. aegypti* and *Culex quinquefasciatus*. Sakthivadivel and Daniel [18] reported in their study that the crude petroleum extract of *T. procumbens* has larvicidal activity against *Culex quinquefasciatus* with LC_50_ > 200 ppm and median mortality against *A. aegypti* and *Anopheles stephensi*.

However, in recent studies on the larvicidal activity of EO components against mosquito species, monoterpenes such as *o*-cymene, limonene, *β*-pinene, (-)-*β*-pinene, *α*-terpinene, *β*-terpinene, and thymol, which are compounds found in the sample of *T. procumbens*, are considered compounds with great larvicidal potential, having action on one or more mosquito species [19].

### 3.3. Cytotoxic Activity with *Artemia salina*

EO to *A. salina* toxicity was performed to preliminarily assess the toxicity of the substances. The data in Table 3 demonstrate the mortality of *A. salina* at different concentrations.

The data in Table 2 show 6.6% mortality at the concentration of 1000 *μ*g mL^−1^ in EO. In addition, with LC_50_ equal to 1238.67 *μ*g mL^−1^, it indicates low toxicity to *A. salina*. This value is above the toxicity standard of potassium dichromate with an LC_50_ of 12.60 *μ*g mL^−1^.

It is possible to identify that EO had no toxicity in the test performed with *A. salina* larvae. This can be reaffirmed by a study that classifies the essential oils in degrees of toxicity and according to the interval, in which the extracts with LC_50_ below 100 *μ*g mL^−1^ are considered to have high toxicity; for moderate activity, the extracts present LC_50_ between 500 and 1000 *μ*g mL^−1^, and for low toxicity, the extracts present LC_50_ above 1000 *μ*g mL^−1^ and are considered nontoxic [10]. In this sense, the EO of the species *T. procumbens* with LC50 = 1238.67 *μ*g mL−1 can be considered as nontoxic.

In this sense, there are papers in the literature that show a good correlation between the toxicity tests on this species and their applicability in different biological activities, such as antifungal, antimicrobial, antitumor, among others, favoring interest in this species in future studies.

This thought resembles studies that conclude that the species present cytotoxicity, and it demonstrates great potential for diverse biological activities [20].

Because it is a preliminary analysis, the low toxicity to *A. salina* may represent that the species present low toxicity to the environment and to the human being, being able to become a promising larvicidal agent.

### 3.4. Antioxidant Activity

The method of sequestration of the DPPH radical is among the several ways to evaluate the antioxidant potential of natural products. This antioxidant activity is measured by the consumption of DPPH; the higher the intake of DPPH in a sample, the lower the IC_50_ and the greater its activity.

The results obtained after determination of the antioxidant activity of EO at different concentrations are shown in Table 4.

Equation of the antioxidant activity of OE: *Y* = 0.2190*x* + 7.4022.

The EO achieved its maximum antioxidant activity (67.1%) at a concentration of 250 *μ*g mL^−1^ with the coefficient of determination (*R*^2^) = 0.9347 and *p* value equal to 0.0016. These data demonstrate that OE presented significant antioxidant activity at its highest concentration.

The EO showed antioxidant activity with an IC_50_ equal to 194.51 *μ*g mL^−1^. Although OE presents compounds such as *β*-karyophylene and thymol that have identified antioxidant activity in the literature, in this study, OE showed low activity when compared to the ascorbic acid pattern. The study by Pereira et al. [21] showed that variations in the content of the indole alkaloids coronaridin and voacangin in the extracts of *T. catharinensis* did not alter the antioxidant potential in vitro, while Chizzola et al. [22] verified high antioxidant properties in the extracts and EO of the leaves of *Thymus vulgaris*, which presented a greater amount of thymol in their constituents.

The isolated action of *β*-caryophyllene or synergism among the major constituents of EO may also be related to the antioxidant activity identified in the study [23].

*T. procumbens* essential oils showed antioxidant activity, reducing levels of oxidative stress when using the DPPH assay. These essential oils appear to have higher antioxidant activity than ascorbic acid and in the study, it is pointed out that increasing the concentration of the essential oil consequently increases the antioxidant power. In the literature, *T. procumbens* is identified as a good candidate for the treatment of inflammation and cancer with fewer toxic effects [24], but these claims are not adequately researched and documented.

For example, *T. procumbens* has been shown to reduce inflammation when applied as a leaf plaster and has been shown to be effective in the treatment of neuropathic and inflammatory pain in rodent models [25]. However, much attention has been given to the antioxidant properties of the plant extracts and the identification of the compounds responsible for these activities. There are two studies that sought to evaluate the elimination of free radicals from *T. procumbens* extract of methanol and ethanol using DPPH and ABTS. In both assays, the extracts showed moderate free radical scavenging activity. The methanol extract showed higher antioxidant activity when compared to the 70% ethanol extract. The effects of the extracts were less potent compared to positive controls with ascorbic acid [26]. The isolated compounds exhibited a very mild effect on the free radicals in both assays.

For example, *T. procumbens* has been shown to reduce inflammation when applied as a leaf plaster and has been shown to be effective in the treatment of neuropathic and inflammatory pain in rodent models [27]. However, much attention has been given to the antioxidant properties of the plant extracts and the identification of the compounds responsible for these activities. There are two studies that sought to evaluate the elimination of free radicals from *T. procumbens* extract of methanol and ethanol using DPPH and ABTS. In both assays, the extracts showed moderate free radical scavenging activity. The methanol extract showed higher antioxidant activity when compared to the 70% ethanol extract. The effects of the extracts were less potent compared to positive controls with ascorbic acid [28]. The isolated compounds exhibited a very mild effect on the free radicals in both assays.

## 4. Conclusion

The chemical composition of *T. procumbens* essential oil indicated the presence of 20 compounds in total. The major compounds are thymol, *γ*-terpinene, and *o*-cymene in its composition. The EO presented antioxidant activity by the DPPH radical capture method at the highest concentrations; however, it presents low activity when compared to ascorbic acid. It also presented low toxicity to *A. salina*, serving as a preliminary analysis of the possible low toxicity of the species to the environment and to humans.

With these results, it is possible to observe that the EO presents as a great promising natural larvicidal agent for use in *A. aegypti* larvae growth sites, necessitating studies of technological bases for the development of finished products and evaluation of toxicity in humans.

The data show the importance of preliminary bioassays as a screening of the biological potential of plant products, as well as the importance of these products as a source of bioactive compounds and demonstrating the biological potential of this species.

## Figures and Tables

**Table 1:** Substances identified in GC-MS analysis of *T. procumbens* essential oil.

No	*t*_*R*_ (min)	IK∗	Compounds	Relative percentage
1	6.328	1002	*α*-Phellandrene	1.33
2	8.307	990	*β*-Myrcene	1.82
3	9.338	1001	(+)-2-carene	2.19
4	9.713	1022	*o*-Cymene	10.27
5	9.812	1024	Limonene	0.38
6	9.944	1026	1,8-Cineole	2.37
7	11.068	1054	*γ*-Terpinene	15.93
8	11.407	1098	Sabinene hydrate	0.62
9	16.129	1177	Terpinen-4-ol	1.22
10	16.317	1171	Umbellulone	0.53
11	18.378	1244	Carvacrol methyl ether	7.70
12	21.921	1299	Thymol	48,22
13	23.432	1352	Thymol acetate	2,56
14	26.549	1424	*β*-Caryophyllene	2.75
15	27.981	1454	*α*-Humulene	0.53
16	28.980	1489	Butylated hydroxyanisole	0.51
17	33.034	1583	Caryophyllene oxide	1.09

IR = retention index of Van den Dool and Kratz (1963); *t*_*R*_ = retention time. Identification of the compounds was performed by the mass spectrum (MS) of the library software LanSolutions GC-MS solution version 2.50 SU1 (NIST05 and WILEY'S Library of Mass Spectra, 9th Edition).

**Table 2:** Percentage mortality (%) of *A. aegypti* larvae at different concentrations of essential oil of *T. procumbens* leaves at 24 and 48 hours.

Concentrations (*µ*g.mL^−1^)	Larvicidal activity (%)	
	24 h	48 h
Control (−)	0	0
Control (+)	100	100
20	6.6	13.32
40	13.32	21.32
60	40	45.2
80	41.2	45.2
100	60	65.2

**Table 3:** Percent mortality of *A. salina* larvae of *T. procumbens* oil at different concentrations.

Oil concentrations (*µ*g.mL^−1^)	Mortality %
50	0
100	0
250	0
500	0
750	0
1000	6.6
LC50	1238.67 *µ*g.mL^−1^
LC(K_2_Cr_2_O_7_)	12.60 *μ*g mL^−1^

**Table 4:** Mean and standard deviation of the percentage of antioxidant activity of *T. procumbens* essential oil in different concentrations.

Oil concentrations (*µ*g.mL^−1^)	Antioxidant activity %	
	Essential oil	Ascorbic acid
7.81	13.0 ± 1.63	18.57 ± 0.52
15.62	13.9 ± 0.46	30 ± 0.10
31.25	14.0 ± 0.38	99.93 ± 0.02
62.5	18.9 ± 0.70	99.99 ± 0.0
125	25.3 ± 0.24	99.99 ± 0.0
250	67.1 ± 0.14	99.99 ± 0.0
IC_50_	194.51 *µ*g mL^−1^	16.71 *µ*g mL^−1^

## Data Availability

The authors declare for the proper purposes that qualitative (phytochemical analysis) and quantitative (antioxidant activity, microbiological activity, cytotoxicity, and larvicidal activity) data used to support the conclusions of this study are included in this article.
